# Supplementary material for: Modeling glioblastoma heterogeneity as a dynamic network of cell states
Source: Mol Syst Biol. 2021 Sep 16;17(9):e10105. doi: 10.15252/msb.202010105 (PMC8444284; doi:10.15252/msb.202010105)
Supplement: Supplementary file 6 — Source Data for Figure 5 [file MSB-17-e10105-s004.zip › Figure5A_sourcedata/GSEA_3017/nosigdb_stateF.GseaPreranked.1622795015644/RICHARDS_CAHOY_JNEUROSCI_2015_ASTRO_IN_VIVO.html]

Details for gene set RICHARDS\_CAHOY\_JNEUROSCI\_2015\_ASTRO\_IN\_VIVO[GSEA]

|  || Dataset | state33017 |
| Phenotype | NoPhenotypeAvailable |
| Upregulated in class | na\_pos |
| GeneSet | RICHARDS\_CAHOY\_JNEUROSCI\_2015\_ASTRO\_IN\_VIVO |
| Enrichment Score (ES) | 0.38646522 |
| Normalized Enrichment Score (NES) | 3.0683765 |
| Nominal p-value | 0.0 |
| FDR q-value | 0.0 |
| FWER p-Value | 0.0 |
Table: GSEA Results Summary

  

Fig 1: Enrichment plot: RICHARDS\_CAHOY\_JNEUROSCI\_2015\_ASTRO\_IN\_VIVO      
 Profile of the Running ES Score & Positions of GeneSet Members on the Rank Ordered List

  

| PROBE | GENE SYMBOL | GENE\_TITLE | RANK IN GENE LIST | RANK METRIC SCORE | RUNNING ES | CORE ENRICHMENT || 1 | CLU |  |  | 5 | 1.852 | 0.0072 | Yes |
| 2 | SPARCL1 |  |  | 6 | 1.804 | 0.0169 | Yes |
| 3 | ATP1B2 |  |  | 9 | 1.759 | 0.0253 | Yes |
| 4 | HEPACAM |  |  | 13 | 1.356 | 0.0310 | Yes |
| 5 | APOE |  |  | 17 | 1.244 | 0.0360 | Yes |
| 6 | S100B |  |  | 20 | 1.167 | 0.0412 | Yes |
| 7 | RAMP1 |  |  | 24 | 1.069 | 0.0453 | Yes |
| 8 | SLC1A3 |  |  | 26 | 1.058 | 0.0505 | Yes |
| 9 | APLP1 |  |  | 28 | 1.047 | 0.0556 | Yes |
| 10 | CSPG5 |  |  | 30 | 1.044 | 0.0606 | Yes |
| 11 | ITPR2 |  |  | 34 | 1.024 | 0.0645 | Yes |
| 12 | DKK3 |  |  | 37 | 0.994 | 0.0688 | Yes |
| 13 | MXD4 |  |  | 39 | 0.987 | 0.0735 | Yes |
| 14 | SLC4A4 |  |  | 40 | 0.980 | 0.0788 | Yes |
| 15 | F3 |  |  | 41 | 0.978 | 0.0841 | Yes |
| 16 | EDNRB |  |  | 44 | 0.957 | 0.0881 | Yes |
| 17 | SAT1 |  |  | 45 | 0.957 | 0.0933 | Yes |
| 18 | CPEB4 |  |  | 46 | 0.953 | 0.0984 | Yes |
| 19 | CXCL14 |  |  | 51 | 0.931 | 0.1012 | Yes |
| 20 | SQSTM1 |  |  | 54 | 0.914 | 0.1050 | Yes |
| 21 | SESN3 |  |  | 56 | 0.908 | 0.1094 | Yes |
| 22 | MYO6 |  |  | 62 | 0.859 | 0.1113 | Yes |
| 23 | SLC1A2 |  |  | 63 | 0.857 | 0.1159 | Yes |
| 24 | TSC22D4 |  |  | 64 | 0.855 | 0.1205 | Yes |
| 25 | SRGAP3 |  |  | 66 | 0.850 | 0.1245 | Yes |
| 26 | GLUL |  |  | 69 | 0.837 | 0.1279 | Yes |
| 27 | GPR155 |  |  | 70 | 0.832 | 0.1324 | Yes |
| 28 | AGT |  |  | 72 | 0.820 | 0.1363 | Yes |
| 29 | CHPT1 |  |  | 73 | 0.818 | 0.1407 | Yes |
| 30 | PDE4DIP |  |  | 82 | 0.794 | 0.1406 | Yes |
| 31 | AQP4 |  |  | 84 | 0.789 | 0.1443 | Yes |
| 32 | CBR1 |  |  | 87 | 0.779 | 0.1474 | Yes |
| 33 | UBL3 |  |  | 88 | 0.778 | 0.1516 | Yes |
| 34 | C1QTNF6 |  |  | 91 | 0.766 | 0.1546 | Yes |
| 35 | LRP1 |  |  | 92 | 0.765 | 0.1587 | Yes |
| 36 | PON2 |  |  | 93 | 0.764 | 0.1628 | Yes |
| 37 | LHFPL3 |  |  | 98 | 0.755 | 0.1647 | Yes |
| 38 | RNF180 |  |  | 101 | 0.751 | 0.1676 | Yes |
| 39 | HES5 |  |  | 102 | 0.749 | 0.1717 | Yes |
| 40 | GRIA2 |  |  | 105 | 0.743 | 0.1746 | Yes |
| 41 | LTBP1 |  |  | 108 | 0.741 | 0.1775 | Yes |
| 42 | CHN1 |  |  | 112 | 0.730 | 0.1798 | Yes |
| 43 | BTG2 |  |  | 124 | 0.708 | 0.1776 | Yes |
| 44 | ZNF106 |  |  | 129 | 0.701 | 0.1792 | Yes |
| 45 | SCG3 |  |  | 130 | 0.698 | 0.1829 | Yes |
| 46 | FEZ1 |  |  | 133 | 0.691 | 0.1856 | Yes |
| 47 | FCGR2A |  |  | 134 | 0.688 | 0.1893 | Yes |
| 48 | DNAJB9 |  |  | 135 | 0.688 | 0.1930 | Yes |
| 49 | CADM2 |  |  | 137 | 0.687 | 0.1961 | Yes |
| 50 | NCAN |  |  | 138 | 0.686 | 0.1998 | Yes |
| 51 | GNAO1 |  |  | 141 | 0.685 | 0.2024 | Yes |
| 52 | DDB2 |  |  | 143 | 0.682 | 0.2055 | Yes |
| 53 | TSPAN7 |  |  | 148 | 0.671 | 0.2070 | Yes |
| 54 | KCNE4 |  |  | 150 | 0.666 | 0.2100 | Yes |
| 55 | TTYH3 |  |  | 154 | 0.655 | 0.2119 | Yes |
| 56 | ITM2C |  |  | 160 | 0.649 | 0.2126 | Yes |
| 57 | PEG3 |  |  | 162 | 0.645 | 0.2156 | Yes |
| 58 | MAPT |  |  | 163 | 0.644 | 0.2190 | Yes |
| 59 | APC |  |  | 169 | 0.632 | 0.2197 | Yes |
| 60 | ATP2B1 |  |  | 170 | 0.632 | 0.2231 | Yes |
| 61 | DAAM1 |  |  | 175 | 0.618 | 0.2243 | Yes |
| 62 | DTNA |  |  | 176 | 0.617 | 0.2276 | Yes |
| 63 | PBX1 |  |  | 179 | 0.613 | 0.2298 | Yes |
| 64 | SSBP2 |  |  | 187 | 0.604 | 0.2292 | Yes |
| 65 | FUT8 |  |  | 194 | 0.593 | 0.2291 | Yes |
| 66 | HERPUD1 |  |  | 197 | 0.590 | 0.2312 | Yes |
| 67 | KLHL24 |  |  | 200 | 0.589 | 0.2333 | Yes |
| 68 | ATP2A2 |  |  | 210 | 0.582 | 0.2315 | Yes |
| 69 | AKAP9 |  |  | 213 | 0.579 | 0.2336 | Yes |
| 70 | DDIT4 |  |  | 216 | 0.579 | 0.2356 | Yes |
| 71 | GPR37L1 |  |  | 220 | 0.576 | 0.2371 | Yes |
| 72 | BTG1 |  |  | 222 | 0.574 | 0.2396 | Yes |
| 73 | PDLIM5 |  |  | 223 | 0.572 | 0.2427 | Yes |
| 74 | KCNJ16 |  |  | 227 | 0.569 | 0.2441 | Yes |
| 75 | HTRA1 |  |  | 229 | 0.568 | 0.2466 | Yes |
| 76 | TMEM132A |  |  | 231 | 0.568 | 0.2491 | Yes |
| 77 | GABARAPL2 |  |  | 232 | 0.568 | 0.2522 | Yes |
| 78 | MEGF10 |  |  | 240 | 0.560 | 0.2514 | Yes |
| 79 | CA2 |  |  | 243 | 0.558 | 0.2533 | Yes |
| 80 | PHYHIPL |  |  | 245 | 0.557 | 0.2557 | Yes |
| 81 | OMG |  |  | 252 | 0.550 | 0.2554 | Yes |
| 82 | TFRC |  |  | 270 | 0.537 | 0.2491 | Yes |
| 83 | HMGCS1 |  |  | 271 | 0.537 | 0.2520 | Yes |
| 84 | LFNG |  |  | 275 | 0.530 | 0.2532 | Yes |
| 85 | PCDH9 |  |  | 278 | 0.527 | 0.2549 | Yes |
| 86 | SLC25A18 |  |  | 280 | 0.525 | 0.2572 | Yes |
| 87 | LRRTM2 |  |  | 281 | 0.524 | 0.2600 | Yes |
| 88 | FMN2 |  |  | 287 | 0.519 | 0.2601 | Yes |
| 89 | MRAS |  |  | 289 | 0.518 | 0.2623 | Yes |
| 90 | CTSF |  |  | 301 | 0.510 | 0.2591 | Yes |
| 91 | BCAN |  |  | 302 | 0.510 | 0.2618 | Yes |
| 92 | PTPRZ1 |  |  | 310 | 0.505 | 0.2607 | Yes |
| 93 | PPP2R2B |  |  | 311 | 0.505 | 0.2634 | Yes |
| 94 | GSTK1 |  |  | 312 | 0.503 | 0.2662 | Yes |
| 95 | NRP2 |  |  | 313 | 0.503 | 0.2689 | Yes |
| 96 | ACYP2 |  |  | 317 | 0.502 | 0.2699 | Yes |
| 97 | PCMTD2 |  |  | 319 | 0.501 | 0.2721 | Yes |
| 98 | HES1 |  |  | 320 | 0.501 | 0.2748 | Yes |
| 99 | RORB |  |  | 328 | 0.497 | 0.2736 | Yes |
| 100 | PSAT1 |  |  | 329 | 0.496 | 0.2763 | Yes |
| 101 | GPC6 |  |  | 332 | 0.495 | 0.2779 | Yes |
| 102 | ACSBG1 |  |  | 345 | 0.490 | 0.2740 | Yes |
| 103 | SEC11C |  |  | 346 | 0.489 | 0.2766 | Yes |
| 104 | GSTM3 |  |  | 348 | 0.488 | 0.2787 | Yes |
| 105 | SERINC1 |  |  | 351 | 0.486 | 0.2802 | Yes |
| 106 | SYT11 |  |  | 352 | 0.486 | 0.2828 | Yes |
| 107 | PSPH |  |  | 359 | 0.484 | 0.2822 | Yes |
| 108 | PDCD4 |  |  | 360 | 0.484 | 0.2848 | Yes |
| 109 | ABAT |  |  | 362 | 0.483 | 0.2868 | Yes |
| 110 | ID2 |  |  | 366 | 0.483 | 0.2878 | Yes |
| 111 | RAP2B |  |  | 372 | 0.480 | 0.2876 | Yes |
| 112 | HSPA5 |  |  | 373 | 0.480 | 0.2902 | Yes |
| 113 | GATM |  |  | 375 | 0.480 | 0.2922 | Yes |
| 114 | SEMA6A |  |  | 381 | 0.478 | 0.2921 | Yes |
| 115 | NEO1 |  |  | 385 | 0.476 | 0.2930 | Yes |
| 116 | TGFB2 |  |  | 390 | 0.474 | 0.2934 | Yes |
| 117 | CASK |  |  | 392 | 0.473 | 0.2954 | Yes |
| 118 | ANK2 |  |  | 394 | 0.472 | 0.2974 | Yes |
| 119 | SORL1 |  |  | 402 | 0.469 | 0.2961 | Yes |
| 120 | FCGR2B |  |  | 404 | 0.468 | 0.2981 | Yes |
| 121 | SLC22A17 |  |  | 412 | 0.465 | 0.2968 | Yes |
| 122 | PSAP |  |  | 418 | 0.463 | 0.2965 | Yes |
| 123 | DPP6 |  |  | 421 | 0.462 | 0.2979 | Yes |
| 124 | SOX5 |  |  | 423 | 0.462 | 0.2999 | Yes |
| 125 | NME3 |  |  | 424 | 0.461 | 0.3023 | Yes |
| 126 | GLG1 |  |  | 433 | 0.459 | 0.3005 | Yes |
| 127 | RPS6KA5 |  |  | 438 | 0.456 | 0.3007 | Yes |
| 128 | LAMP1 |  |  | 440 | 0.455 | 0.3026 | Yes |
| 129 | TRPS1 |  |  | 441 | 0.455 | 0.3051 | Yes |
| 130 | SPRY4 |  |  | 443 | 0.453 | 0.3070 | Yes |
| 131 | MAST4 |  |  | 446 | 0.451 | 0.3083 | Yes |
| 132 | DDIT3 |  |  | 447 | 0.451 | 0.3107 | Yes |
| 133 | APC2 |  |  | 449 | 0.450 | 0.3126 | Yes |
| 134 | LONRF2 |  |  | 451 | 0.449 | 0.3145 | Yes |
| 135 | TTYH1 |  |  | 457 | 0.448 | 0.3142 | Yes |
| 136 | BTBD1 |  |  | 459 | 0.447 | 0.3160 | Yes |
| 137 | CROT |  |  | 460 | 0.447 | 0.3184 | Yes |
| 138 | VPS13C |  |  | 468 | 0.445 | 0.3170 | Yes |
| 139 | NRXN2 |  |  | 469 | 0.444 | 0.3194 | Yes |
| 140 | TIPARP |  |  | 472 | 0.444 | 0.3207 | Yes |
| 141 | ZNF521 |  |  | 476 | 0.442 | 0.3215 | Yes |
| 142 | ATXN2 |  |  | 477 | 0.442 | 0.3238 | Yes |
| 143 | STK38L |  |  | 482 | 0.440 | 0.3240 | Yes |
| 144 | RFTN2 |  |  | 485 | 0.439 | 0.3253 | Yes |
| 145 | RAB9A |  |  | 489 | 0.438 | 0.3260 | Yes |
| 146 | TMEM30A |  |  | 490 | 0.438 | 0.3284 | Yes |
| 147 | GJA1 |  |  | 495 | 0.436 | 0.3285 | Yes |
| 148 | FAT3 |  |  | 501 | 0.434 | 0.3282 | Yes |
| 149 | FGD4 |  |  | 503 | 0.434 | 0.3299 | Yes |
| 150 | TIMP4 |  |  | 505 | 0.433 | 0.3317 | Yes |
| 151 | ATP6V0D1 |  |  | 512 | 0.430 | 0.3308 | Yes |
| 152 | ATP6AP2 |  |  | 514 | 0.430 | 0.3325 | Yes |
| 153 | FGFR3 |  |  | 515 | 0.429 | 0.3349 | Yes |
| 154 | EYA4 |  |  | 525 | 0.424 | 0.3322 | Yes |
| 155 | ELOVL2 |  |  | 527 | 0.424 | 0.3340 | Yes |
| 156 | PELI2 |  |  | 529 | 0.422 | 0.3357 | Yes |
| 157 | ANTXR1 |  |  | 537 | 0.418 | 0.3341 | Yes |
| 158 | TMEM9B |  |  | 539 | 0.417 | 0.3358 | Yes |
| 159 | TANC2 |  |  | 542 | 0.417 | 0.3370 | Yes |
| 160 | RASSF2 |  |  | 544 | 0.417 | 0.3387 | Yes |
| 161 | PFKM |  |  | 547 | 0.416 | 0.3398 | Yes |
| 162 | ANKRA2 |  |  | 550 | 0.415 | 0.3410 | Yes |
| 163 | PNPLA8 |  |  | 551 | 0.414 | 0.3432 | Yes |
| 164 | GPM6B |  |  | 553 | 0.413 | 0.3449 | Yes |
| 165 | PPP3CA |  |  | 554 | 0.413 | 0.3471 | Yes |
| 166 | KCNN2 |  |  | 556 | 0.412 | 0.3488 | Yes |
| 167 | PAPSS1 |  |  | 560 | 0.411 | 0.3493 | Yes |
| 168 | BICD1 |  |  | 563 | 0.410 | 0.3505 | Yes |
| 169 | HDAC9 |  |  | 579 | 0.404 | 0.3445 | Yes |
| 170 | SLC6A1 |  |  | 582 | 0.404 | 0.3456 | Yes |
| 171 | CTSO |  |  | 584 | 0.403 | 0.3472 | Yes |
| 172 | FOS |  |  | 592 | 0.401 | 0.3455 | Yes |
| 173 | PLEKHB1 |  |  | 597 | 0.399 | 0.3455 | Yes |
| 174 | ALKBH7 |  |  | 606 | 0.396 | 0.3433 | Yes |
| 175 | RND2 |  |  | 608 | 0.396 | 0.3449 | Yes |
| 176 | LSAMP |  |  | 611 | 0.394 | 0.3459 | Yes |
| 177 | SLC40A1 |  |  | 612 | 0.393 | 0.3480 | Yes |
| 178 | SASH1 |  |  | 614 | 0.393 | 0.3496 | Yes |
| 179 | TMTC2 |  |  | 619 | 0.392 | 0.3495 | Yes |
| 180 | NTRK3 |  |  | 621 | 0.391 | 0.3511 | Yes |
| 181 | FKBP2 |  |  | 622 | 0.391 | 0.3532 | Yes |
| 182 | RCN2 |  |  | 623 | 0.391 | 0.3553 | Yes |
| 183 | TLCD1 |  |  | 625 | 0.390 | 0.3568 | Yes |
| 184 | CHST2 |  |  | 628 | 0.390 | 0.3578 | Yes |
| 185 | TAOK3 |  |  | 631 | 0.389 | 0.3588 | Yes |
| 186 | SEMA6D |  |  | 637 | 0.388 | 0.3582 | Yes |
| 187 | PINK1 |  |  | 639 | 0.387 | 0.3597 | Yes |
| 188 | ATP6V1H |  |  | 642 | 0.387 | 0.3607 | Yes |
| 189 | THRA |  |  | 643 | 0.386 | 0.3628 | Yes |
| 190 | TMEM14A |  |  | 647 | 0.386 | 0.3633 | Yes |
| 191 | GPRC5B |  |  | 653 | 0.385 | 0.3626 | Yes |
| 192 | SEZ6L |  |  | 657 | 0.383 | 0.3630 | Yes |
| 193 | GPHN |  |  | 658 | 0.383 | 0.3651 | Yes |
| 194 | ID4 |  |  | 659 | 0.383 | 0.3671 | Yes |
| 195 | DOCK4 |  |  | 668 | 0.380 | 0.3648 | Yes |
| 196 | CDH10 |  |  | 669 | 0.379 | 0.3669 | Yes |
| 197 | ADD3 |  |  | 674 | 0.378 | 0.3667 | Yes |
| 198 | BAZ2B |  |  | 675 | 0.377 | 0.3688 | Yes |
| 199 | ASTN1 |  |  | 679 | 0.375 | 0.3691 | Yes |
| 200 | RAB6B |  |  | 688 | 0.374 | 0.3668 | Yes |
| 201 | ZFYVE21 |  |  | 694 | 0.373 | 0.3661 | Yes |
| 202 | PTPRN2 |  |  | 705 | 0.369 | 0.3626 | Yes |
| 203 | CLIP3 |  |  | 710 | 0.369 | 0.3624 | Yes |
| 204 | VLDLR |  |  | 713 | 0.368 | 0.3633 | Yes |
| 205 | LRP8 |  |  | 719 | 0.366 | 0.3626 | Yes |
| 206 | PFN2 |  |  | 724 | 0.365 | 0.3624 | Yes |
| 207 | TAGLN3 |  |  | 725 | 0.365 | 0.3643 | Yes |
| 208 | ZDHHC21 |  |  | 728 | 0.364 | 0.3652 | Yes |
| 209 | PCSK2 |  |  | 731 | 0.363 | 0.3661 | Yes |
| 210 | SNN |  |  | 732 | 0.363 | 0.3680 | Yes |
| 211 | PPP1R14C |  |  | 733 | 0.363 | 0.3700 | Yes |
| 212 | WNT7A |  |  | 735 | 0.362 | 0.3714 | Yes |
| 213 | SC5D |  |  | 737 | 0.362 | 0.3728 | Yes |
| 214 | GAL3ST4 |  |  | 738 | 0.362 | 0.3747 | Yes |
| 215 | FAS |  |  | 740 | 0.361 | 0.3761 | Yes |
| 216 | ZHX3 |  |  | 741 | 0.361 | 0.3781 | Yes |
| 217 | MARK1 |  |  | 757 | 0.357 | 0.3718 | Yes |
| 218 | FGF1 |  |  | 771 | 0.353 | 0.3666 | Yes |
| 219 | FLCN |  |  | 772 | 0.353 | 0.3685 | Yes |
| 220 | LRP4 |  |  | 773 | 0.353 | 0.3704 | Yes |
| 221 | PTPRA |  |  | 777 | 0.352 | 0.3707 | Yes |
| 222 | COX7C |  |  | 778 | 0.352 | 0.3726 | Yes |
| 223 | TTC3 |  |  | 779 | 0.352 | 0.3745 | Yes |
| 224 | MSI2 |  |  | 780 | 0.352 | 0.3764 | Yes |
| 225 | RORA |  |  | 783 | 0.351 | 0.3772 | Yes |
| 226 | SLC15A2 |  |  | 784 | 0.351 | 0.3791 | Yes |
| 227 | PSD3 |  |  | 788 | 0.349 | 0.3793 | Yes |
| 228 | HSD17B7 |  |  | 791 | 0.349 | 0.3801 | Yes |
| 229 | C3orf70 |  |  | 794 | 0.348 | 0.3809 | Yes |
| 230 | RDH5 |  |  | 798 | 0.347 | 0.3811 | Yes |
| 231 | CADPS2 |  |  | 802 | 0.346 | 0.3813 | Yes |
| 232 | KCTD12 |  |  | 808 | 0.345 | 0.3805 | Yes |
| 233 | FCGRT |  |  | 814 | 0.344 | 0.3796 | Yes |
| 234 | EMID1 |  |  | 816 | 0.344 | 0.3809 | Yes |
| 235 | PPARGC1A |  |  | 821 | 0.343 | 0.3806 | Yes |
| 236 | OSBPL1A |  |  | 828 | 0.341 | 0.3791 | Yes |
| 237 | TRAF3IP2 |  |  | 829 | 0.341 | 0.3810 | Yes |
| 238 | DAG1 |  |  | 834 | 0.340 | 0.3806 | Yes |
| 239 | VEGFA |  |  | 840 | 0.339 | 0.3797 | Yes |
| 240 | NUDT3 |  |  | 842 | 0.339 | 0.3810 | Yes |
| 241 | RHOQ |  |  | 843 | 0.339 | 0.3828 | Yes |
| 242 | RFX3 |  |  | 844 | 0.338 | 0.3847 | Yes |
| 243 | PCDHGC3 |  |  | 845 | 0.337 | 0.3865 | Yes |
| 244 | ZFP36L2 |  |  | 850 | 0.336 | 0.3861 | No |
| 245 | NKD1 |  |  | 858 | 0.334 | 0.3841 | No |
| 246 | FGF12 |  |  | 873 | 0.331 | 0.3782 | No |
| 247 | CDS2 |  |  | 879 | 0.330 | 0.3773 | No |
| 248 | STAT3 |  |  | 880 | 0.330 | 0.3791 | No |
| 249 | SPIRE1 |  |  | 881 | 0.329 | 0.3808 | No |
| 250 | WSB1 |  |  | 882 | 0.329 | 0.3826 | No |
| 251 | MAPK4 |  |  | 886 | 0.328 | 0.3827 | No |
| 252 | CRY1 |  |  | 890 | 0.327 | 0.3829 | No |
| 253 | DST |  |  | 896 | 0.327 | 0.3819 | No |
| 254 | SCAMP1 |  |  | 900 | 0.326 | 0.3820 | No |
| 255 | TMCC1 |  |  | 902 | 0.326 | 0.3832 | No |
| 256 | BACE1 |  |  | 907 | 0.324 | 0.3828 | No |
| 257 | ADCYAP1R1 |  |  | 913 | 0.324 | 0.3818 | No |
| 258 | NCAM2 |  |  | 915 | 0.324 | 0.3830 | No |
| 259 | GDPD2 |  |  | 921 | 0.323 | 0.3820 | No |
| 260 | TMOD2 |  |  | 930 | 0.321 | 0.3794 | No |
| 261 | CADM1 |  |  | 935 | 0.320 | 0.3790 | No |
| 262 | UNC13B |  |  | 936 | 0.320 | 0.3807 | No |
| 263 | CLCN3 |  |  | 937 | 0.320 | 0.3824 | No |
| 264 | MARCH6 |  |  | 941 | 0.319 | 0.3825 | No |
| 265 | NPAS2 |  |  | 955 | 0.317 | 0.3771 | No |
| 266 | TNRC6B |  |  | 965 | 0.316 | 0.3739 | No |
| 267 | HIF1A |  |  | 972 | 0.314 | 0.3723 | No |
| 268 | AGL |  |  | 976 | 0.314 | 0.3724 | No |
| 269 | HIBADH |  |  | 979 | 0.313 | 0.3730 | No |
| 270 | ARID4A |  |  | 984 | 0.312 | 0.3725 | No |
| 271 | ALDH6A1 |  |  | 987 | 0.311 | 0.3731 | No |
| 272 | TLE2 |  |  | 993 | 0.311 | 0.3720 | No |
| 273 | FMNL2 |  |  | 1005 | 0.309 | 0.3677 | No |
| 274 | RGMA |  |  | 1010 | 0.308 | 0.3672 | No |
| 275 | EFNB3 |  |  | 1020 | 0.307 | 0.3639 | No |
| 276 | PCMTD1 |  |  | 1026 | 0.305 | 0.3628 | No |
| 277 | SERPINE2 |  |  | 1030 | 0.304 | 0.3628 | No |
| 278 | SCRG1 |  |  | 1032 | 0.304 | 0.3639 | No |
| 279 | ATRN |  |  | 1037 | 0.302 | 0.3634 | No |
| 280 | DEAF1 |  |  | 1042 | 0.302 | 0.3628 | No |
| 281 | CTNND2 |  |  | 1048 | 0.300 | 0.3617 | No |
| 282 | PELI1 |  |  | 1059 | 0.298 | 0.3579 | No |
| 283 | SORBS1 |  |  | 1068 | 0.297 | 0.3551 | No |
| 284 | LINGO1 |  |  | 1074 | 0.297 | 0.3540 | No |
| 285 | TMEM68 |  |  | 1075 | 0.296 | 0.3556 | No |
| 286 | DHRS7 |  |  | 1079 | 0.296 | 0.3556 | No |
| 287 | SPECC1 |  |  | 1089 | 0.294 | 0.3522 | No |
| 288 | LYPD1 |  |  | 1094 | 0.293 | 0.3516 | No |
| 289 | RTN3 |  |  | 1095 | 0.293 | 0.3532 | No |
| 290 | SCARA3 |  |  | 1096 | 0.293 | 0.3548 | No |
| 291 | DNER |  |  | 1104 | 0.292 | 0.3526 | No |
| 292 | KALRN |  |  | 1106 | 0.292 | 0.3536 | No |
| 293 | PIK3R1 |  |  | 1121 | 0.289 | 0.3475 | No |
| 294 | LAMA2 |  |  | 1123 | 0.289 | 0.3485 | No |
| 295 | SLC7A11 |  |  | 1131 | 0.288 | 0.3463 | No |
| 296 | FBXL17 |  |  | 1136 | 0.287 | 0.3456 | No |
| 297 | B3GAT1 |  |  | 1145 | 0.286 | 0.3428 | No |
| 298 | KCND2 |  |  | 1151 | 0.285 | 0.3416 | No |
| 299 | GLRB |  |  | 1161 | 0.284 | 0.3383 | No |
| 300 | SEPT8 |  |  | 1167 | 0.283 | 0.3371 | No |
| 301 | GDPD1 |  |  | 1178 | 0.282 | 0.3331 | No |
| 302 | MACF1 |  |  | 1179 | 0.282 | 0.3346 | No |
| 303 | CNR1 |  |  | 1180 | 0.282 | 0.3362 | No |
| 304 | ZBTB20 |  |  | 1181 | 0.281 | 0.3377 | No |
| 305 | SLC35A5 |  |  | 1184 | 0.281 | 0.3381 | No |
| 306 | TRPM3 |  |  | 1187 | 0.280 | 0.3385 | No |
| 307 | NAP1L5 |  |  | 1188 | 0.280 | 0.3400 | No |
| 308 | METRNL |  |  | 1196 | 0.279 | 0.3377 | No |
| 309 | RGS20 |  |  | 1197 | 0.279 | 0.3392 | No |
| 310 | FNDC4 |  |  | 1203 | 0.279 | 0.3380 | No |
| 311 | JAM2 |  |  | 1204 | 0.279 | 0.3395 | No |
| 312 | ADAM17 |  |  | 1215 | 0.277 | 0.3355 | No |
| 313 | LGR4 |  |  | 1216 | 0.277 | 0.3370 | No |
| 314 | KLHL5 |  |  | 1226 | 0.276 | 0.3336 | No |
| 315 | CA8 |  |  | 1230 | 0.276 | 0.3335 | No |
| 316 | TMEM175 |  |  | 1234 | 0.276 | 0.3333 | No |
| 317 | CDH11 |  |  | 1240 | 0.275 | 0.3321 | No |
| 318 | STARD4 |  |  | 1254 | 0.274 | 0.3265 | No |
| 319 | ALDH7A1 |  |  | 1255 | 0.273 | 0.3279 | No |
| 320 | VCAM1 |  |  | 1266 | 0.272 | 0.3240 | No |
| 321 | SALL2 |  |  | 1274 | 0.271 | 0.3216 | No |
| 322 | KIF1B |  |  | 1276 | 0.271 | 0.3225 | No |
| 323 | ST8SIA5 |  |  | 1277 | 0.271 | 0.3240 | No |
| 324 | SLC6A9 |  |  | 1278 | 0.271 | 0.3254 | No |
| 325 | LMBRD1 |  |  | 1280 | 0.271 | 0.3264 | No |
| 326 | LENG8 |  |  | 1284 | 0.270 | 0.3262 | No |
| 327 | NLGN3 |  |  | 1292 | 0.269 | 0.3238 | No |
| 328 | PGM2L1 |  |  | 1295 | 0.269 | 0.3242 | No |
| 329 | ARHGEF7 |  |  | 1299 | 0.268 | 0.3240 | No |
| 330 | SSR1 |  |  | 1300 | 0.268 | 0.3254 | No |
| 331 | SMOX |  |  | 1305 | 0.268 | 0.3247 | No |
| 332 | ADCY9 |  |  | 1306 | 0.268 | 0.3261 | No |
| 333 | IRF2BP2 |  |  | 1311 | 0.267 | 0.3254 | No |
| 334 | RFX4 |  |  | 1320 | 0.266 | 0.3225 | No |
| 335 | C4orf19 |  |  | 1326 | 0.265 | 0.3212 | No |
| 336 | GLUD1 |  |  | 1334 | 0.264 | 0.3188 | No |
| 337 | TTC14 |  |  | 1336 | 0.263 | 0.3196 | No |
| 338 | MAPK8IP1 |  |  | 1340 | 0.263 | 0.3194 | No |
| 339 | PRRX1 |  |  | 1341 | 0.262 | 0.3208 | No |
| 340 | ELAVL4 |  |  | 1358 | 0.261 | 0.3135 | No |
| 341 | PCDH8 |  |  | 1360 | 0.261 | 0.3144 | No |
| 342 | TMEM63B |  |  | 1370 | 0.260 | 0.3109 | No |
| 343 | SLCO1C1 |  |  | 1372 | 0.260 | 0.3118 | No |
| 344 | NRXN1 |  |  | 1374 | 0.259 | 0.3126 | No |
| 345 | ARHGEF12 |  |  | 1379 | 0.259 | 0.3118 | No |
| 346 | SLC27A1 |  |  | 1380 | 0.259 | 0.3132 | No |
| 347 | ABCD3 |  |  | 1381 | 0.259 | 0.3146 | No |
| 348 | ABHD3 |  |  | 1382 | 0.259 | 0.3160 | No |
| 349 | CTSA |  |  | 1385 | 0.258 | 0.3163 | No |
| 350 | GEM |  |  | 1391 | 0.257 | 0.3150 | No |
| 351 | PDE4B |  |  | 1404 | 0.256 | 0.3098 | No |
| 352 | OPHN1 |  |  | 1405 | 0.256 | 0.3112 | No |
| 353 | MEIS1 |  |  | 1407 | 0.256 | 0.3120 | No |
| 354 | LBH |  |  | 1410 | 0.255 | 0.3123 | No |
| 355 | DCHS1 |  |  | 1411 | 0.255 | 0.3137 | No |
| 356 | PDHB |  |  | 1413 | 0.255 | 0.3145 | No |
| 357 | GLDC |  |  | 1425 | 0.255 | 0.3099 | No |
| 358 | PTAR1 |  |  | 1441 | 0.254 | 0.3031 | No |
| 359 | ACO2 |  |  | 1443 | 0.253 | 0.3039 | No |
| 360 | NHSL1 |  |  | 1459 | 0.251 | 0.2971 | No |
| 361 | PCDH19 |  |  | 1460 | 0.251 | 0.2984 | No |
| 362 | SLC33A1 |  |  | 1461 | 0.251 | 0.2998 | No |
| 363 | DNAJB14 |  |  | 1465 | 0.251 | 0.2995 | No |
| 364 | JUNB |  |  | 1466 | 0.251 | 0.3008 | No |
| 365 | APLP2 |  |  | 1468 | 0.250 | 0.3016 | No |
| 366 | BBS2 |  |  | 1472 | 0.250 | 0.3014 | No |
| 367 | CUL3 |  |  | 1483 | -0.253 | 0.2973 | No |
| 368 | C2orf69 |  |  | 1500 | -0.259 | 0.2900 | No |
| 369 | ITGB1BP1 |  |  | 1501 | -0.259 | 0.2914 | No |
| 370 | BRD8 |  |  | 1508 | -0.263 | 0.2895 | No |
| 371 | TDG |  |  | 1509 | -0.263 | 0.2909 | No |
| 372 | ACAT2 |  |  | 1512 | -0.263 | 0.2912 | No |
| 373 | HCFC1 |  |  | 1515 | -0.264 | 0.2916 | No |
| 374 | PURB |  |  | 1527 | -0.267 | 0.2870 | No |
| 375 | JTB |  |  | 1556 | -0.273 | 0.2733 | No |
| 376 | PSMD11 |  |  | 1572 | -0.278 | 0.2666 | No |
| 377 | CD320 |  |  | 1577 | -0.279 | 0.2659 | No |
| 378 | FUBP1 |  |  | 1578 | -0.280 | 0.2674 | No |
| 379 | SRRM2 |  |  | 1587 | -0.283 | 0.2646 | No |
| 380 | ZFP36L1 |  |  | 1590 | -0.284 | 0.2650 | No |
| 381 | ENAH |  |  | 1592 | -0.284 | 0.2660 | No |
| 382 | PRKDC |  |  | 1594 | -0.285 | 0.2670 | No |
| 383 | LHX2 |  |  | 1597 | -0.285 | 0.2675 | No |
| 384 | USP7 |  |  | 1636 | -0.295 | 0.2484 | No |
| 385 | APOLD1 |  |  | 1648 | -0.299 | 0.2440 | No |
| 386 | MTDH |  |  | 1651 | -0.302 | 0.2445 | No |
| 387 | GSPT1 |  |  | 1653 | -0.303 | 0.2456 | No |
| 388 | UBE2D3 |  |  | 1669 | -0.306 | 0.2391 | No |
| 389 | BZW2 |  |  | 1671 | -0.306 | 0.2402 | No |
| 390 | KLHL23 |  |  | 1674 | -0.308 | 0.2408 | No |
| 391 | BRD9 |  |  | 1675 | -0.309 | 0.2424 | No |
| 392 | FADS1 |  |  | 1682 | -0.312 | 0.2408 | No |
| 393 | THRAP3 |  |  | 1698 | -0.318 | 0.2344 | No |
| 394 | FARP1 |  |  | 1706 | -0.321 | 0.2323 | No |
| 395 | PHLDA1 |  |  | 1718 | -0.324 | 0.2281 | No |
| 396 | UGP2 |  |  | 1725 | -0.327 | 0.2266 | No |
| 397 | UPP1 |  |  | 1726 | -0.328 | 0.2283 | No |
| 398 | TOP1 |  |  | 1749 | -0.340 | 0.2182 | No |
| 399 | GLO1 |  |  | 1755 | -0.342 | 0.2173 | No |
| 400 | PPP2R5E |  |  | 1827 | -0.378 | 0.1807 | No |
| 401 | PTCH1 |  |  | 1857 | -0.393 | 0.1670 | No |
| 402 | ACTL6A |  |  | 1859 | -0.393 | 0.1686 | No |
| 403 | SQLE |  |  | 1863 | -0.396 | 0.1691 | No |
| 404 | CHD7 |  |  | 1874 | -0.400 | 0.1658 | No |
| 405 | SFPQ |  |  | 1908 | -0.429 | 0.1502 | No |
| 406 | EIF5 |  |  | 1923 | -0.439 | 0.1449 | No |
| 407 | SERBP1 |  |  | 1938 | -0.447 | 0.1397 | No |
| 408 | STAG1 |  |  | 1943 | -0.450 | 0.1399 | No |
| 409 | TPRKB |  |  | 1944 | -0.450 | 0.1424 | No |
| 410 | RBM3 |  |  | 1995 | -0.487 | 0.1178 | No |
| 411 | FABP5 |  |  | 2033 | -0.523 | 0.1005 | No |
| 412 | IER2 |  |  | 2034 | -0.524 | 0.1033 | No |
| 413 | CKLF |  |  | 2055 | -0.544 | 0.0953 | No |
| 414 | GGH |  |  | 2080 | -0.603 | 0.0855 | No |
| 415 | CKAP5 |  |  | 2086 | -0.623 | 0.0861 | No |
| 416 | H2AFV |  |  | 2143 | -0.779 | 0.0598 | No |
Table: GSEA details [plain text format]

  

Fig 2: RICHARDS\_CAHOY\_JNEUROSCI\_2015\_ASTRO\_IN\_VIVO: Random ES distribution      
 Gene set null distribution of ES for **RICHARDS\_CAHOY\_JNEUROSCI\_2015\_ASTRO\_IN\_VIVO**
